# Chicken PIAS2 enhances H6N2 avian influenza virus replication by promoting SUMOylation of viral NP

**DOI:** 10.1186/s13567-025-01702-w

**Published:** 2025-12-18

**Authors:** Junsheng Zhang, Qian Xue, Xiyi Wang, Huixing Yang, Minfan Huang, Meng Zhang, Luxiang Zhao, Wenqing Wang, Zuxian Chen, Peirong Jiao

**Affiliations:** 1 College of Veterinary Medicine, South China Agricultural University, 483 Wushan Road, Tianhe District, Guangzhou, 510642 China; 2 Key Laboratory of Animal Vaccine Development, Ministry of Agriculture and Rural Affairs, Guangzhou, China; 3 Guangdong Provincial Key Laboratory of Zoonosis Prevention and Control, Guangzhou, China

**Keywords:** Chicken, protein inhibitor of activated STAT2, H6N2 avian influenza virus, nucleoprotein, SUMOylation

## Abstract

Avian influenza virus (AIV) is a significant zoonotic pathogen that causes infectious disease in various species and poses a serious threat to both the poultry industry and public health. Mammalian protein inhibitors of activated STAT2 (PIAS2) have been shown to affect viral replication by interacting with viral proteins. However, the role of chicken PIAS2 (chPIAS2) in regulating H6N2 subtype AIV replication remains unclear. In this study, we cloned chPIAS2 from primary chicken embryo fibroblast cells and identified that it contains five conserved domains. Overexpression of chPIAS2 promoted the replication of H6N2 AIV, although chPIAS2 expression was also induced during viral infection. We further found that chPIAS2 interacted with the H6N2 AIV nucleoprotein (NP) (DK65-NP) in the nucleus. ChPIAS2 promoted the SUMOylation of DK65-NP through its SUMO E3 ligase activity. We also confirmed that the lysine residues at positions 7, 48, 77, and 113 are the small ubiquitin-like modifier (SUMO) conjugation (SUMOylation) sites of DK65-NP. Collectively, our results indicate that chPIAS2 promotes H6N2 AIV replication by enhancing the SUMOylation of viral NP. In conclusion, our study reveals the role of chPIAS2 in AIV replication and provides new insights into the molecular mechanisms underlying AIV pathogenicity in poultry, suggesting a potential therapeutic target for avian influenza.

## Introduction

Small ubiquitin-like modifier (SUMO) is a protein involved in SUMO conjugation (SUMOylation), a reversible post-translational modification characterized by the covalent attachment of SUMO to the lysine residues on target proteins or by its interaction with SUMO-interacting motif (SIM)-containing domains on target proteins [1]. This modification process involves an E1 activating enzyme, an E2 conjugating enzyme (Ubc9), and SUMO E3 ligases [1]. SUMOylation plays a role in the cell cycle, transcription, DNA repair, antiviral immunity, and viral replication by altering the stability, folding, and protein–protein interactions of target proteins [2]. Therefore, SUMOylation serves as a potential therapeutic target for cancer, myocardial infarction, glioblastoma, brain ischemia, atherosclerosis, tumors, and other metabolic, infectious, and inflammation-related diseases [3–8]. SUMO E3 ligases include the protein inhibitor of activated STAT (PIAS) family, RanBP2, and Pc2 [9–11]. PIAS proteins regulate diverse cellular processes by promoting the SUMOylation of target proteins [12]. PIAS proteins can serve as bona fide therapeutic targets to inhibit the STAT pathway in cancers, such as urothelial cancer [13, 14]. PIAS1 is also considered a therapeutic target for inflammatory bowel disease through its regulation of NF-κB-mediated inflammatory signaling [15]. Mammalian PIAS proteins are encoded by four genes, *PIAS1*, *PIAS2* (also called *PIASx*), *PIAS3*, and *PIAS4* (also called *PIASy*) [12]. Human PIAS2 has two isoforms, PIASxα and PIASxβ. However, whether PIAS genes exist in poultry remains unclear.

Several studies have shown that human PIASxα interacts with influenza A virus nucleoprotein (NP) to regulate the intracellular trafficking of NP and elevate viral production [16]. Human PIAS1 binds to the Epstein–Barr virus Rta protein and human Ubc9 to increase the SUMOylation and transactivation activity of the Rta protein [17]. PIASxβ preferentially enhances the SUMOylation of the Epstein–Barr virus Rta protein [18]. Human PIAS2 restricts hepatitis C virus replication by regulating the SUMOylation of the viral core protein [19]. Therefore, there is evidence suggesting that mammalian PIAS proteins are involved in regulating viral replication and proliferation.

Avian influenza viruses (AIVs) are the most common infectious respiratory tract pathogens, which cause infection in poultry, humans, pigs, and cattle, and they pose a serious threat to both the animal industry and public health [20]. Low pathogenic avian influenza viruses (LPAIVs) cause mild clinical disease in poultry. However, some H6 subtype LPAIVs have reassorted with other AIV subtypes to generate highly pathogenic avian influenza viruses (HPAIVs), posing a clear threat to human health [21]. Nevertheless, the mechanism by which chPIAS2 regulates H6N2 LPAIV replication remains unclear.

Here, we found that chPIAS2 promotes SUMOylation of the H6N2 LPAIV NP through its SUMO E3 ligase activity, thereby enhancing viral replication. In summary, these findings provide new insights into the molecular mechanisms underlying the pathogenicity of AIVs in poultry and suggest a potential therapeutic target for AIV infection.

## Materials and methods

### Virus and cells

An H6N2 subtype AIV, A/duck/Guangdong/65/2008 (referred to as DK65), was isolated from ducks in Guangdong, China, in 2008. The purified H6N2 virus was propagated in 9-day-old specific-pathogen-free embryonated chicken eggs. Human embryonic kidney 293 T (293 T) cells, DF-1 cells, and primary chicken embryo fibroblast cells were cultured in Dulbecco’s modified Eagle medium (DMEM) (Gibco, USA) supplemented with 10% fetal bovine serum (FBS) (Gibco, USA), 100 U/mL penicillin, and 100 μg/mL streptomycin (Gibco, USA), and incubated at 37 °C in a humidified atmosphere containing 5% (v/v) CO_2_.

### Construction of expression plasmids

The *DK65-NP* gene (GenBank accession no. PV963174 [22]) was cloned into the eukaryotic expression vector pCAGGS to construct the expression plasmid DK65-NP-Flag using standard molecular biology techniques. The coding sequence of chicken PIAS2 (chPIAS2) (GenBank accession no. PV975640) was amplified from chicken complementary DNA (cDNA) using standard molecular biology techniques. The functional domains of chPIAS2 were predicted using the Simple Modular Architecture Research Tool [23]. A phylogenetic tree was constructed with MEGA-X software using the neighbor-joining method. The *chPIAS2* gene was cloned into pCAGGS to generate the chPIAS2-HA expression plasmid. DK65-NP mutants in which lysine (K) residues were replaced with arginine (R) were cloned into pCAGGS using overlap polymerase chain reaction (PCR). Plasmids encoding chicken SUMO1 (chSUMO1) (GenBank accession no. PV975641), chicken SENP1 (chSENP1) (GenBank accession no. PV975643), and chicken Ubc9 (chUbc9) (GenBank accession no. PV975642) were stored in our laboratory. All plasmids were confirmed via sequencing. The primer sequences are presented in Table 1.

**Table 1:** **Sequences of primers used in this study**

Primer	Sequence (5′–3′)
chPIAS2-HA-F1	CGGAATTCATGGCGGATTTCGAGGAGCTGC
chPIAS2-HA-R1	GACAGGACAGATCGCGGTAGGCTTCTTTTC
chPIAS2-HA-F2	GAAAAGAAGCCTACCGCGATCTGTCCTGTC
chPIAS2-HA-R2	CCGCTCGAGTCAAGCGTAGTCTGGGACGTCGTATGGGTAGTCCAGTGAGATGATGTCA
DK65NP-C-FLAG-F	GGGAATTCATGGCGTCTCAAGGCACC
DK65NP-C-FLAG-R	CCGCTCGAGTTACTTATCGTCGTCATCCTTGTAATCATTGTCATACTCCTCTGCAT
DK65NP-K7R-F1	GGGAATTCATGGCGTCTCAAGGCACCAGACGATC
DK65NP-K7R-R1	CCGCTCGAGTTACTTATCGTCGTCATCCTTGTAATCATTGTCATACTCCTC
DK65NP-K48R-F1	GGGAATTCATGGCGTCTCAAGGCACC
DK65NP-K48R-R1	CATAGTCGCTGAGTCTGAGTTCAGT
DK65NP-K48R-F2	ACTGAACTCAGACTCAGCGACTATG
DK65NP-K48R-R2	CCGCTCGAGTTACTTATCGTCGTCATCCTTGTAATCATTGTCATACTCCTC
DK65NP-K77R-R1	CAGGTATCTGTTCCTCCTTTCA
DK65NP-K77R-F2	TGAAAGGAGGAACAGATACCTG
DK65NP-K87R-R1	CTTGGGTCTCTTCCTGCATT
DK65NP-K87R-F2	AATGCAGGAAGAGACCCAAG
DK65NP-K90R-R1	GACCTCCAGTCTTTCTTGGGTCT
DK65NP-K90R-F2	AGACCCAAGAAAGACTGGAGGTC
DK65NP-K91R-R1	GACCTCCAGTTCTTCTTGGGTCT
DK65NP-K91R-F2	AGACCCAAAGAGAACTGGAGGTC
DK65NP-K103R-R1	CTCTCATCCATCTACCGTCT
DK65NP-K103R-F2	AGACGGTAGATGGATGAGAG
DK65NP-K113R-R1	GATCTCCTCTCTGTCATACAGA
DK65NP-K113R-F2	TCTGTATGACAGAGAGGAGATC
DK65NP-K184R-R1	CCGACTCCTCTTACTGCTGCA
DK65NP-K184R-F2	TGCAGCAGTAAGAGGAGTCGG
DK65NP-K198R-R1	CATTAATCCCTCGTCTTATCAT
DK65NP-K198R-F2	ATGATAAGACGAGGGATTAATG
DK65NP-K227R-R1	GGAATTTCCCTCTGAGGATGTT
DK65NP-K227R-F2	AACATCCTCAGAGGGAAATTCC
DK65NP-K229R-R1	GGAATCTCCCTTTGAGGATGTT
DK65NP-K229R-F2	AACATCCTCAAAGGGAGATTCC
DK65NP-K273R-R1	CAAGCAGGATCTATGGGCCA
DK65NP-K273R-F2	TGGCCCATAGATCCTGCTTG
DK65NP-K325R-R1	CAATTGACTTCTATGTGCAGGA
DK65NP-K325R-F2	TCCTGCACATAGAAGTCAATTG
DK65NP-K470R-R1	GGTTCGTTGCTCTTTCGTCCGA
DK65NP-K470R-F2	TCGGACGAAAGAGCAACGAACC
DK65NP-K483R-R1	CCGCTCGAGTTACTTATCGTCGTCATCCTTGTAATCATTGTCATACTCCTCTGCATTGTCTCCGAAGAAATAAGATCCTTCATTACTCATG
qchPIAS2-F	ACTGTCCTCCTATGTTTTTGG
qchPIAS2-R	AACTCCCGTCTCACTCCTGT
qchβ-actin-F	ACCCCAAAGCCAACAGAGAG
qchβ-actin-R	GATGGGCACAGTGTGGGTAA

### RNA extraction and quantitative real-time PCR

Total RNA from DF-1 cells was extracted using the RNeasy Mini Kit (Promega, USA) and reverse-transcribed into complementary DNA (cDNA) using the M-MLV Kit (Promega, USA), following the manufacturer’s instructions. cDNA from each sample was used to perform quantitative real-time PCR (qRT-PCR) with gene-specific primers (Table 1). The qRT-PCR reactions were conducted using the Bio-Rad CFX96 Touch™ Real-Time PCR Detection System (Bio-Rad, USA). Each reaction mixture contained 1 μL of cDNA, 100 nM of each primer, and 10 μL of 2 × SYBR Green qPCR Master Mix (Promega, USA). Expression levels of target genes were normalized to chicken β-actin using the 2^−ΔΔCt^ method.

### Coimmunoprecipitation assay

Plasmids were transfected into 1 × 10^7^ 293 T cells using Lipofectamine 2000 reagent (Invitrogen, USA) following the manufacturer’s instructions. After 36 h, 293 T cells were lysed in NP-40 lysis buffer (Beyotime Biotechnology, China), supplemented with 1% protease inhibitor cocktail and *N*-ethylmaleimide. After incubation at 4 °C for 30 min, insoluble components were removed via centrifugation at 13800 *g* at 4 °C for 15 min. The lysates were incubated with anti-Flag agarose beads (Invitrogen, USA) at 4 °C for 6 h. The beads were washed three times with cold phosphate-buffered saline (PBS). Precipitated proteins were separated via sodium dodecyl sulfate–polyacrylamide gel electrophoresis (SDS–PAGE) and transferred to nitrocellulose membranes (Merck Millipore Co., Billerica, MA, USA). The membranes were blocked with 5% bovine serum albumin (BSA) in tris-buffered saline with Tween 20 (TBST) (GEN-VIEW Scientific Inc, USA) for 1 h at room temperature, and the target proteins were detected using appropriate primary antibodies (Invitrogen, USA), followed by DyLight 800-conjugated goat anti-mouse IgG (LI-COR, USA). The target proteins were visualized using the Odyssey infrared imaging system (LI-COR, USA).

### Confocal microscopy assay

ChPIAS2-mCherry and DK65-NP-eGFP plasmids were transfected into 293 T and DF-1 cells. After 24 h, cells were fixed with 4% paraformaldehyde at room temperature for 30 min. Cells were then permeabilized with 0.1% Triton X-100 at room temperature for 10 min. Nuclei were counterstained with DAPI, and cells were mounted using 50% glycerol. Fluorescent images were captured using a confocal microscope (Olympus, Japan).

### Viral growth curves

DF-1 cells were seeded in 6-well plates, and chPIAS2 was transfected into the cells. At 24 h post-transfection, the cells were infected with DK65 at a multiplicity of infection equivalent to 1000 times the 50% tissue culture infective dose (TCID_50_). After incubation for 1 h, the virus-containing medium was removed and replaced with DMEM containing 0.05% BSA and 0.01 μg/mL tosyl phenylalanyl chloromethyl ketone (TPCK)-treated trypsin (Sigma-Aldrich, Germany). Cell supernatants were collected separately at 12, 24, and 36 h post-infection. The TCID_50_ of DK65 in the supernatants was determined using the Reed–Muench method [24].

### Statistical analysis

Data are presented as means ± standard deviation (SD). Statistical analysis was performed using GraphPad Prism 8.0.2 software (GraphPad Software, USA). An unpaired two-tailed Student’s *t*-test was used to evaluate the differences between groups. Differences were considered statistically significant at a *p*-value of < 0.05 (^∗^*p* < 0.05; ^∗∗^*p* < 0.01).

## Results

### Cloning and sequencing of chPIAS2

**Figure 1:**
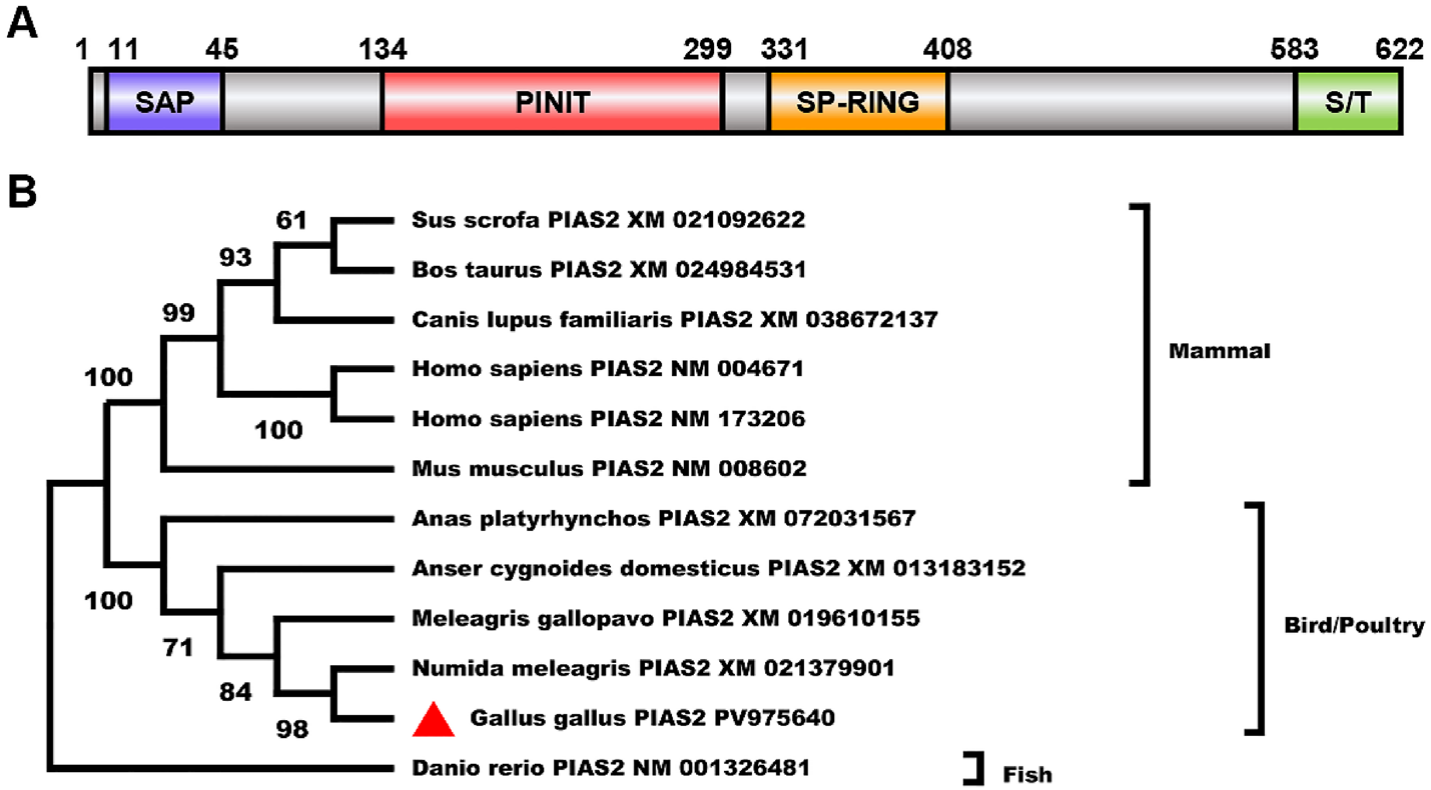
**The molecular characterization of chPIAS2**. **A** The functional domains of chPIAS2. The SAP domain (11–45 aa) is shown in purple, the PINIT domain (134–299 aa) is shown in red, the RLD (RING-finger-like zinc-binding domain) (331–408 aa) is shown in orange, the AD (highly acidic domain) domain is not shown, and the S/T motif is shown in green.** B** The phylogenetic tree of PIAS2 was from different species. A phylogenetic tree was built by MEGA-X software on the basis of the neighbor-joining method.

The PIAS family has been identified in many species, and mammalian PIAS2 is involved in a variety of cellular processes. To determine whether the *PIAS2* gene is present in chickens, we designed primers for chPIAS2 on the basis of *PIAS2* gene sequences from different species available in the GenBank database. Total RNA was extracted from primary chicken embryo fibroblast cells and reverse-transcribed into cDNA to clone chPIAS2. Sequencing results showed that the coding sequence of chPIAS2 (PV975640) is 1869 base pairs (bp) in length, encoding 622 amino acids. The functional domains of chPIAS2 were predicted using SMART software. The analysis revealed that chPIAS2 contains an SAP domain at the N-terminus, as well as a PINIT domain, a RING-finger-like domain (RLD), an AD, a serine/threonine (S/T) motif at the C-terminus, and a SUMO E3 ligase active site located in the RLD of chPIAS2 (Figure 1A). This active site corresponds to a tryptophan (W) residue at position 383 (W383), similar to that found in mammalian PIAS2. The amino acid sequence of chPIAS2 was compared with those from other species using MEGA-X software. The results indicated that the homology of chPIAS2 with duck PIAS2, goose PIAS2, mouse PIAS2, human PIASxα, and PIASxβ was 93.6%, 93.6%, 82.4%, 81.2%, and 82.1%, respectively (Table 2). Phylogenetic analysis showed that chPIAS2 was clustered into the poultry clade (Figure 1B). Collectively, these findings demonstrate that the *PIAS2* gene is present in chickens and possesses conserved functional domains, similar to those in mammals.

**Table 2:** **The amino acid homology of chPIAS2 with different species**

Species	Accession no.	Gene	Homology (%)
*Numida meleagris* (guinea fowl)	XM_021379901	*PIAS2*	97.4
*Anas platyrhynchos* (duck)	XM_072031567	*PIAS2*	93.6
*Anser cygnoides* (goose)	XM_013183152	*PIAS2*	93.6
*Meleagris gallopavo* (turkey)	XM_019610155	*PIAS2*	93.5
*Sus scrofa* (swine)	XM_021092622	*PIAS2*	82.9
*Bos taurus* (cattle)	XM_024984531	*PIAS2*	82.8
*Canis lupus familiaris* (dog)	XM_038672137	*PIAS2*	82.7
*Mus musculus* (mouse)	NM_008602	*PIAS2*	82.4
*Homo sapiens* (human)	NM_004671	*PIASxβ*	82.1
*Homo sapiens* (human)	NM_173206	*PIASxα*	81.2
*Danio rerio* (zebrafish)	XM_001326481	*PIAS2*	68.3

### DK65 infection upregulates chPIAS2 expression

**Figure 2:**
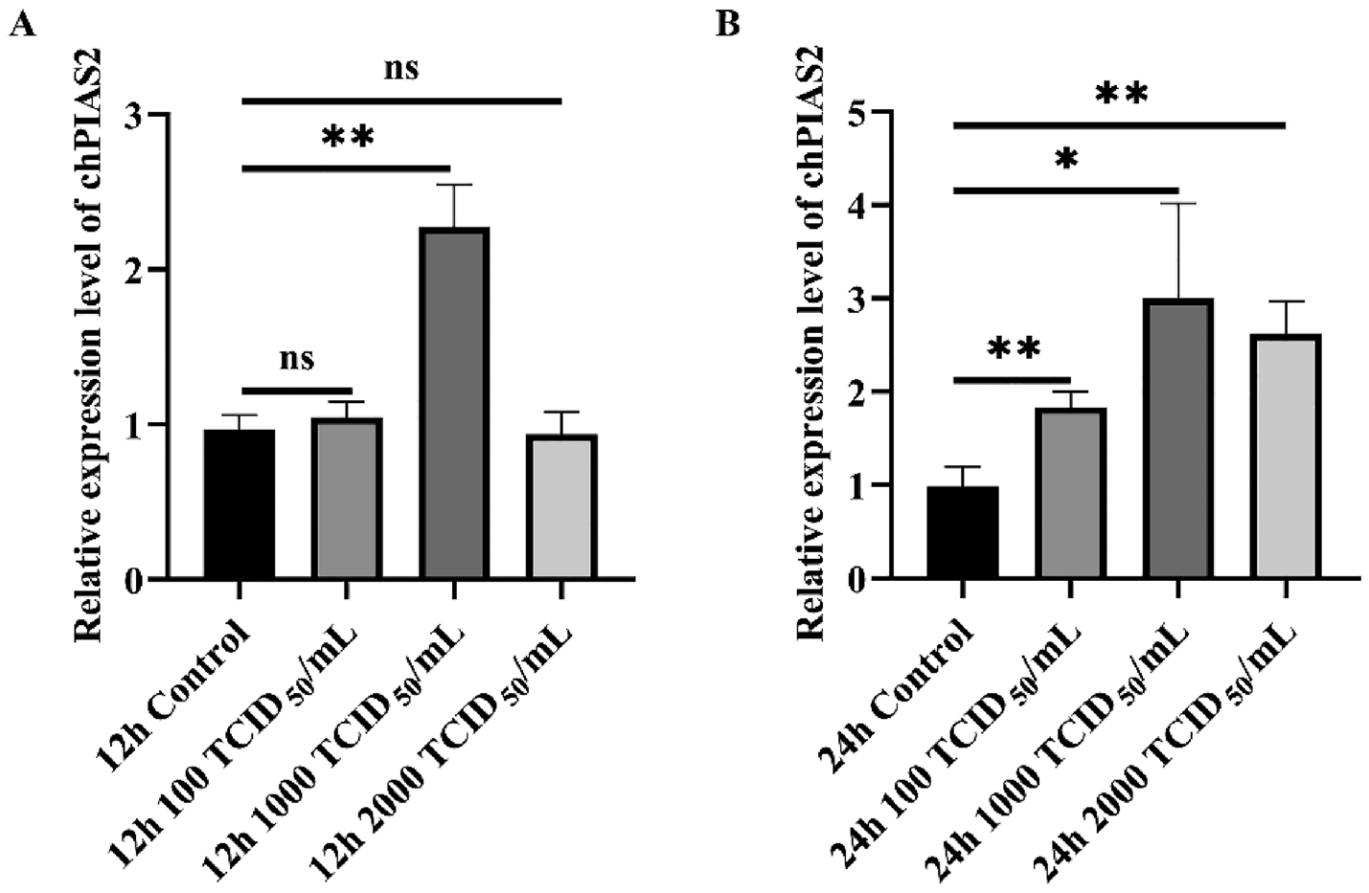
**DK65 infection induces the expression of chPIAS2**. DF-1 cells infected with varying titers (100, 1000, and 2000 TCID_50_/mL) of DK65. **A** After 12 h infection and **B** after 24 h infection, the mRNA expression level of chPIAS2 was measured by qRT-PCR (*p* < 0.01, *p* < 0.01, *p* < 0.05, and *p* < 0.01, respectively). Statistics were analyzed via unpaired two-tailed Student’s *t*-test: nonsignificant (ns) *p* > 0.05; ^*^p < 0.05; and ^**^p < 0.01.

Influenza A virus A/WSN/1933 (WSN, H1N1) infection has been shown to induce the expression of human PIAS1 both in vitro and in vivo [25]. Similarly, H5N1 subtype AIV infection promotes the expression of duck PIAS2 in duck embryo fibroblast cells [26]. To investigate the relationship between chPIAS2 expression and DK65 infection, we measured mRNA expression levels of chPIAS2 in DF-1 cells infected with varying titers (100, 1000, and 2000 TCID_50_/mL) of DK65. As shown in Figure 2A, the mRNA expression level of chPIAS2 in DF-1 cells infected at a dose of 1000 TCID_50_/mL was significantly higher than that in uninfected cells at 12 h post-infection (*p* < 0.01). Similarly, the mRNA expression level of chPIAS2 in cells infected with 100, 1000, and 2000 TCID_50_/mL of the virus was significantly higher than that in uninfected cells at 24 h post-infection (*p* < 0.01, *p* < 0.05, and *p* < 0.01, respectively; Figure 2B). These findings indicate that chPIAS2 expression is upregulated in response to DK65 infection.

### Overexpression of chPIAS2 promotes DK65 replication

**Figure 3:**
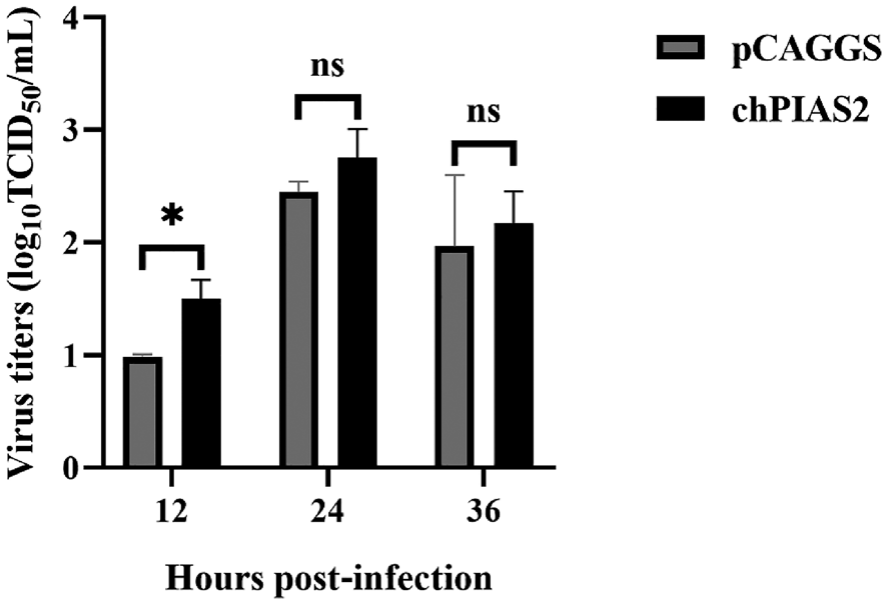
**Overexpression of chPIAS2 promotes DK65 replication**. The chPIAS2 and pCAGGS (expression vector, as control) were transfected into DF-1 cells. The transfected cells were infected with 1000 TCID_50_/mL of DK65 at 24 h post-transfection. The viral titers in the cell supernatants were measured using TCID_50_/mL method at 12, 24, and 36 h post-infection. The viral titers were expressed as means ± SD in log_10_ TCID_50_/mL, and unpaired two-tailed Student’s *t*-test was used to analyze the differences (ns *p* > 0.05; ^*^p < 0.05).

To further investigate the role of chPIAS2 in viral replication, chPIAS2 was transfected into DF-1 cells. After 24 h, the transfected cells were infected with 1000 TCID_50_/mL of DK65. Viral titers in the cell supernatants were measured using the TCID_50_ method at 12, 24, and 36 h post-infection. The result showed that viral titers were significantly increased in chPIAS2-overexpressing DF-1 cells at 12 h post-infection (*p* < 0.05) (Figure 3). These findings indicate that overexpression of chPIAS2 promotes DK65 replication.

### chPIAS2 interacts with DK65-NP in the nucleus

**Figure 4:**
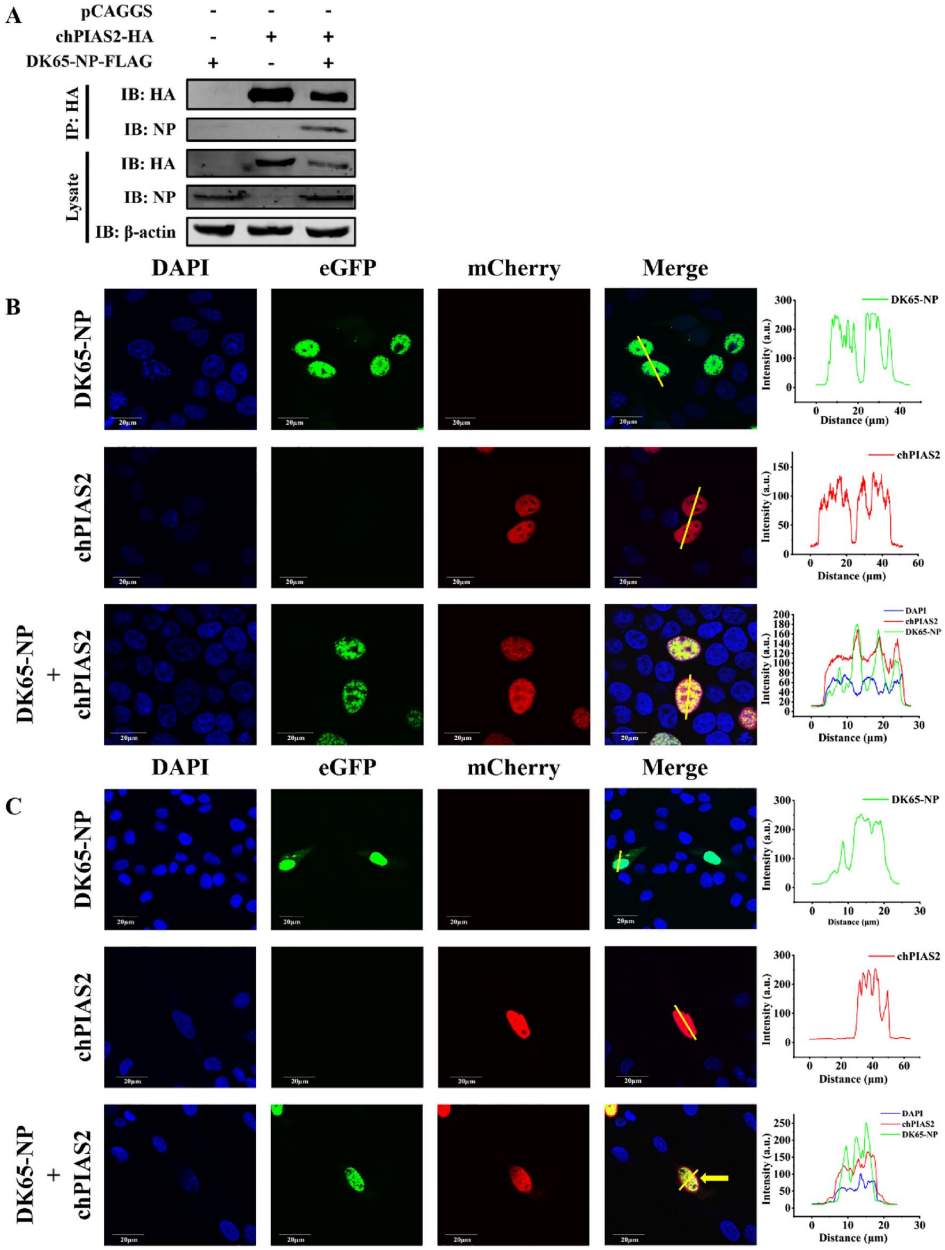
**ChPIAS2 interacts with DK65-NP**. **A** The interaction of chPIAS2 and DK65-NP. 293 T cells were co-transfected with the chPIAS2-HA and DK65-NP-Flag plasmids. After 24 h of transfection, cell lysates were coimmunoprecipitated with HA-tagged agarose, followed by western blot with anti-HA and anti-NP mAbs. **B** The subcellular localization of chPIAS2 and DK65-NP in 293 T cells. **C** The subcellular localization of chPIAS2 and DK65-NP in DF-1 cells. 293 T (**B**) and DF-1 (**C**) cells were transfected with chPIAS2-mCherry and DK65-NP-eGFP plasmids; cells were fixed and permeabilized at 24 h post-transfection. Nuclei were counterstained with DAPI (blue). The chPIAS2 (red) and DK65-NP (green) were visualized using a confocal microscope (Olympus, original magnification, 60×).

In the nucleus, human PIAS1 and the two isoforms of PIAS2 (PIASxα and PIASxβ) have been shown to interact with the Epstein–Barr virus Rta protein, promoting its SUMOylation [17, 18]. PIAS1 also interacts with the Zaire Ebola virus VP35 protein and the human cytomegalovirus (HCMV) IE2 protein [27, 28]. Additionally, human PIAS2 binds to hepatitis C virus core protein [19]. NP, a multifunctional protein encoded by influenza viruses, is a critical component of the viral RNP complex [29]. Human PIAS1 has been shown to bind to NP of H1N1 (WSN) influenza A virus [25]. To investigate whether chPIAS2 interacts with DK65-NP, 293 T cells were co-transfected with the DK65-NP-Flag and chPIAS2-HA plasmids. Cell lysates were collected 24 h post-transfection. Western blot analysis using an anti-Flag antibody revealed that DK65-NP was immunoprecipitated by anti-HA agarose beads (Figure 4A), indicating that chPIAS2 interacts with DK65-NP in 293 T cells.

To further examine the subcellular localization of chPIAS2 and DK65-NP, chPIAS2-mCherry and DK65-NP-eGFP were transfected into 293 T and DF-1 cells, respectively. Confocal laser scanning microscopy assay showed that chPIAS2 localized predominantly in the nucleus, while DK65-NP was distributed in both the cytoplasm and nucleus. Notably, chPIAS2 was colocalized with DK65-NP in the nuclei of 293 T and DF-1 cells (Figures 4B, C).

Taken together, these results demonstrate that chPIAS2 interacts with DK65 NP in the nucleus.

### chPIAS2 enhances the SUMOylation of DK65-NP through its SUMO E3 ligase activity in a reversible manner

**Figure 5:**
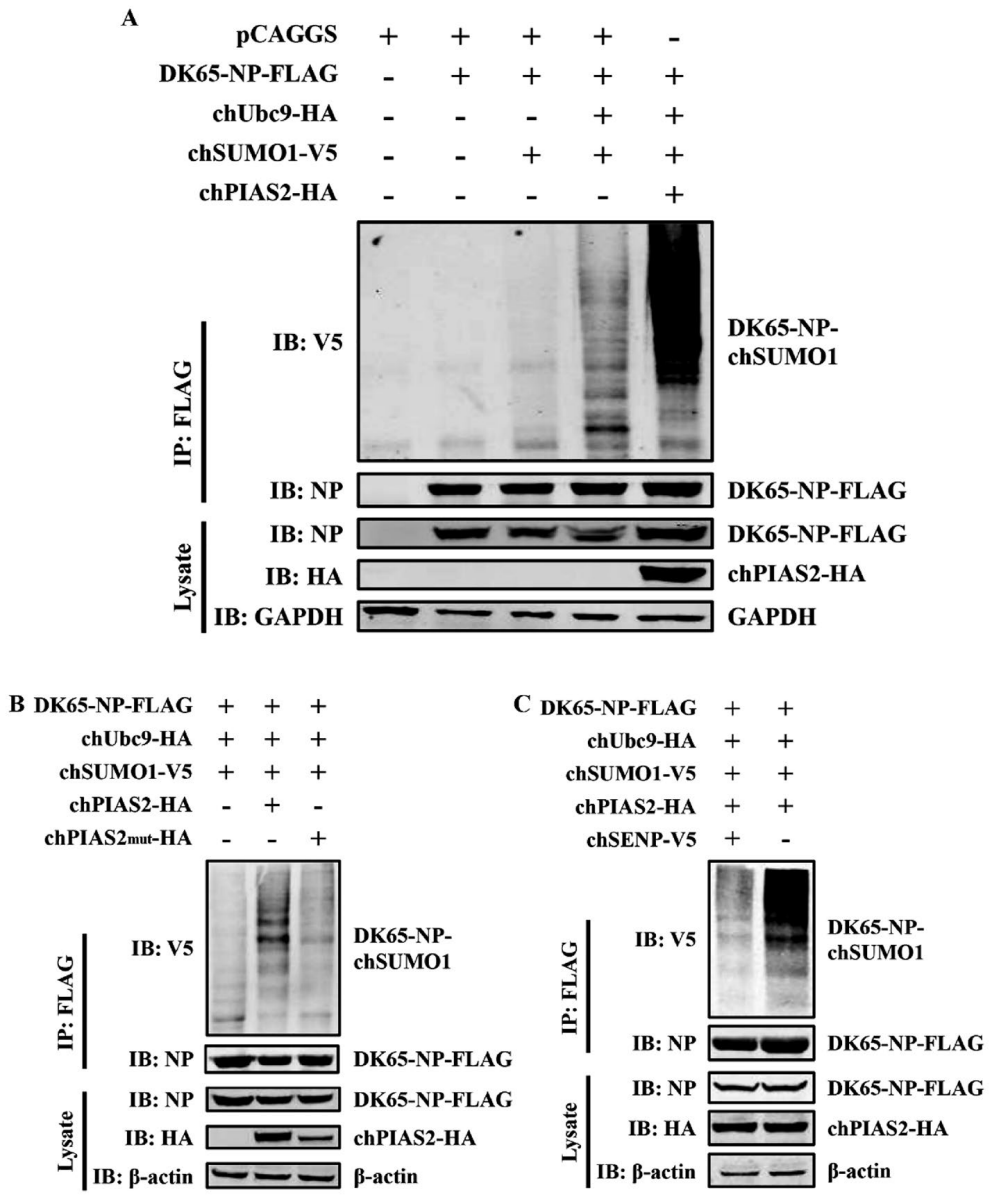
**chPIAS2 enhances the SUMOylation of DK65-NP through its SUMO E3 ligase activity in a reversible manner.** **A** ChPIAS2 enhanced the SUMOylation of DK65-NP. 293 T cells were co-transfected with DK65-NP-Flag, pCAGGS, chUbc9-HA, chSUMO1-V5, and chPIAS2-HA. After 36 h of transfection, cell lysates were coimmunoprecipitated with anti-Flag agarose, and the proteins were detected by western blot. **B** ChPIAS2 increased SUMOylation of DK65-NP through its SUMO E3 ligase activity. ChPIAS2-HA or chPIAS2mut-HA was co-transfected into 293 T cells following DK65-NP-Flag, pCAGGS, chUbc9-HA, and chSUMO1-V5. After 36 h of transfection, cell lysates were coimmunoprecipitated with anti-Flag agarose, and the proteins were detected by western blot. **C** SUMOylation of DK65-NP was reversible. 293 T cells were co-transfected with DK65-NP-Flag, pCAGGS, chUbc9-HA, chSUMO1-V5, chPIAS2-HA, and chSENP1-V5. After 36 h of transfection, cell lysates were coimmunoprecipitated with anti-Flag agarose, and the proteins were detected by western blot with the indicated antibodies.

PIAS2 is a SUMO E3 ligase that promotes the SUMOylation of target proteins. Previous studies have shown that the NP of the WSN strain can be SUMOylated by SUMO1 [16]. In our earlier results, we found that chPIAS2 interacts with DK65-NP in the nucleus. To determine whether chPIAS2 is involved in the SUMOylation of this NP, 293 T cells were co-transfected with DK65-NP-Flag, pCAGGS, chUbc9-HA, chSUMO1-V5, and chPIAS2-HA. Cell lysates were collected 36 h post-transfection. Western blot analysis using an anti-V5 antibody showed that chSUMO1 was immunoprecipitated by anti-Flag agarose beads. Notably, the high molecular-weight bands in cells co-expressing DK65-NP-Flag, chSUMO1-V5, chUbc9-HA, and chPIAS2-HA group were markedly stronger than those in the other groups (Figure 5A). This result suggests that chPIAS2 enhances the SUMOylation of DK65-NP.

The SUMOylation of the HCMV IE2 protein is significantly enhanced by wild-type PIAS1 but not by a PIAS1-Cys351Ser mutant [27]. Human PIASxα_mut_ (W383A), which lacks E3 ligase activity, fails to promote SUMOylation of the androgen receptor [30]. Sequence analysis revealed chPIAS2, similar to human PIASxα and PIASxβ, contains W383. To examine whether chPIAS2 increases SUMOylation of DK65-NP through its SUMO E3 ligase activity, we generated a mutant form of chPIAS2 (chPIAS2mut) with a W383A substitution using site-directed mutagenesis. The wild-type (chPIAS2-HA) or mutant (chPIAS2mut-HA) was co-transfected into 293 T cells with DK65-NP-Flag, chSUMO1-V5, and chUbc9-HA. Cell lysates were collected 36 h post-transfection and subjected to coimmunoprecipitation. Western blot analysis showed that the high molecular-weight forms of DK65-NP were markedly reduced in cells expressing chPIAS2mut-HA compared with those expressing chPIAS2-HA (Figure 5B). These findings confirm that chPIAS2 increases SUMOylation of DK65-NP through its SUMO E3 ligase activity.

SUMOylation is a reversible process regulated by sentrin-specific proteases (SENPs), which deconjugate SUMO moieties from target proteins [31]. To assess whether SUMOylation of DK65-NP is reversible, chSENP1-V5 or pCAGGS were co-transfected into 293 T cells with chUbc9-HA, chSUMO1-V5, chPIAS2-HA, and DK65-NP-Flag. Cell lysates were collected 36 h post-transfection and subjected to coimmunoprecipitation. The high molecular-weight forms of DK65-NP were significantly reduced in the presence of chSENP1-V5 compared with the control group (Figure 5C), indicating that SUMOylation of DK65-NP is reversible.

Taken together, these results demonstrate that chPIAS2 promotes the SUMOylation of DK65-NP through its SUMO E3 ligase activity, and that this modification can be reversed by SENP1.

### Lysine residues K7, K48, K77, and K113 are SUMOylation sites of DK65-NP

**Figure 6:**
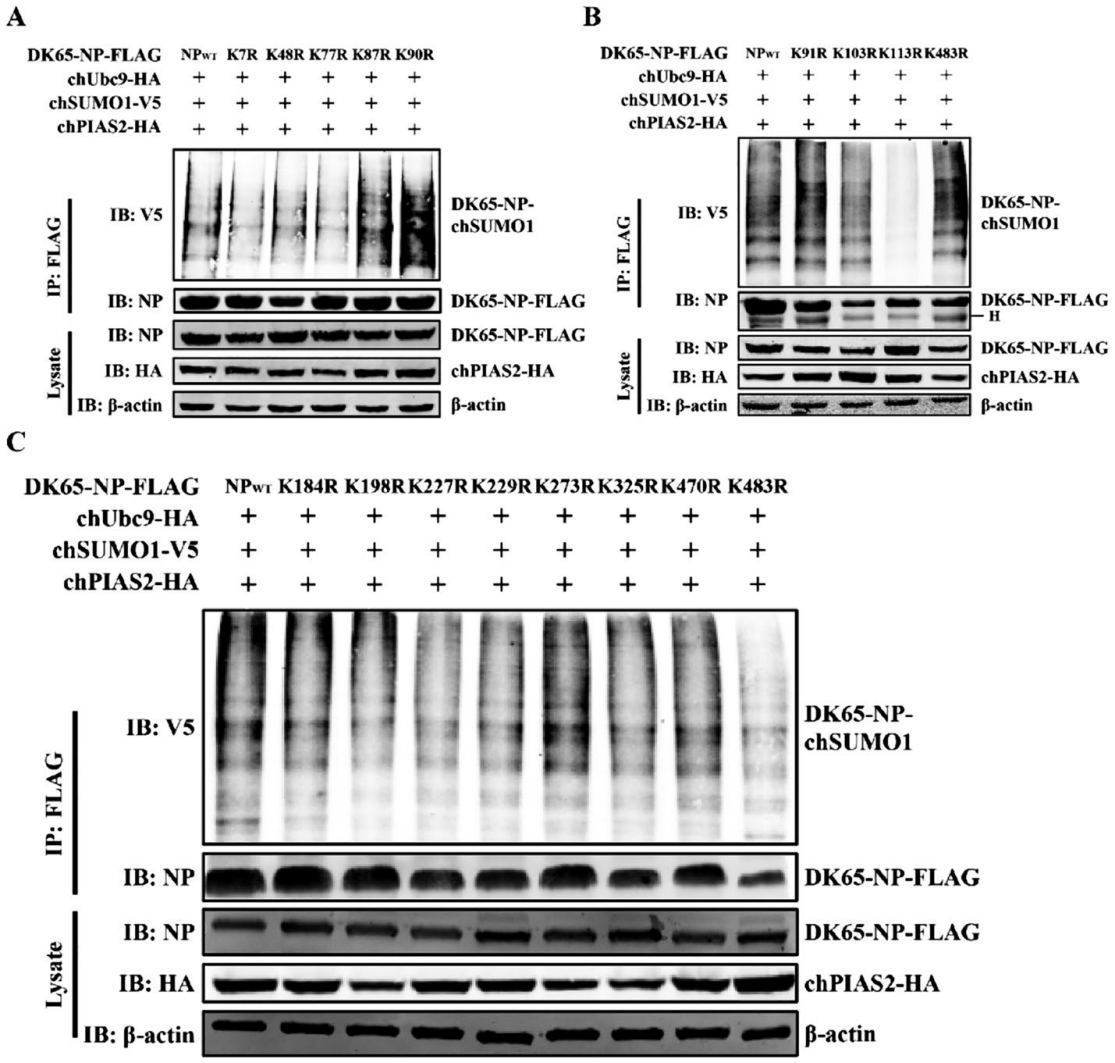
**Lysine residues K7, K48, K77, and K113 are SUMOylation sites of DK65-NP.** Wild-type or mutant DK65-NP constructs were co-transfected with chUbc9-HA, chSUMO1-V5, and chPIAS2-HA in 293 T cells, respectively. Cells lysates were collected 36 h post-transfection and coimmunoprecipitated with anti-Flag agarose, and the proteins were detected by western blot with the indicated antibodies.

SUMOylation typically occurs on lysine (K) residues of target proteins through attachment to the Gly-Gly motif of SUMO [32]. To identify the SUMOylation site(s) on NP of DK65, each lysine residue was individually mutated to arginine (R) via site-directed mutagenesis (Table 1), generating a panel of NP mutants: K7R, K48R, K77R, K87R, K90R, K91R, K103R, K113R, K184R, K198R, K227R, K229R, K273R, K325R, K470R, and K483R. Wild-type or mutant DK65-NP constructs were co-transfected with chUbc9-HA, chSUMO1-V5, and chPIAS2-HA into 293 T cells. Cell lysates were collected 36 h post-transfection and subjected to coimmunoprecipitation. The high molecular-weight forms were absent in the K7R, K48R, K77R, and K113R mutants (Figure 6), indicating that DK65-NP is SUMOylated at these sites (K7, K48, K77, and K113).

Taken together, these results demonstrate that chPIAS2 promotes SUMOylation of DK65-NP at lysine residues K7, K48, K77, and K113 through its SUMO E3 ligase activity.

## Discussion

SUMOylation is a dynamic and reversible post-translational modification that influences protein activity, protein–protein interactions, and the subcellular localization of target proteins [1]. It is widely conserved across various species, including humans, mice, cattle, carp, and fruit flies [1]. As a SUMO E3 ligase, the protein inhibitor of activated STAT (PIAS) family regulates transcription, immune responses, and viral replication by promoting the SUMOylation of target proteins [12]. Mammalian PIAS proteins possess five conserved domains: the SAP domain, PINIT domain, RING-finger-like domain (RLD), AD, and C-terminal S/T domain (with the exception of PIAS4) [12]. The SAP domain recognizes and binds A + T-rich DNA sequences to regulate transcription [33], while the RLD is conserved and essential for SUMO E3 ligase activity of PIAS proteins. The W383A substitution within the RLD abolishes this ligase activity [30]. In our previous study, we cloned duck PIAS2 and found that it contained the characteristic SAP–PINIT–RLD–AD–S/T sequence [34]. However, whether PIAS genes exist in chickens has remained unclear. In this study, we cloned the *chPIAS2* gene from primary chicken embryo fibroblast cells. The *chPIAS2* gene was 1869 bp long and encoded a 622-amino acid protein. The amino acid sequence of chPIAS2 showed 93.6%, 93.6%, 82.4%, 81.2%, and 82.1% similarity with duck PIAS2, goose PIAS2, mouse PIAS2, human PIASxα, and PIASxβ, respectively. Moreover, chPIAS2 contained the SAP, PINIT, RLD, and AD domains, along with the S/T motif, similar to PIAS2 in ducks and mammals. These findings indicate that *PIAS2* is a highly conserved gene across different species; however, whether chPIAS2 functions similarly to mammalian PIAS2 remains to be determined.

In this study, we found that chPIAS2 expression was upregulated in response to H6N2 AIV (DK65) infection. Similarly, we found that H7N9 AIV infection also upregulated the expression of chPIAS2 (data not shown). These results indicated that the upregulation of chPIAS2 was induced by H6N2 and H7N9 subtype AIV infection. The upregulation of human PIAS1 is induced upon influenza A virus infection (WSN (H1N1), AH13 (H7N9), AH05 (H5N1), and SH13 (H9N2)) [25]. Our previous study has shown that H5N1 AIV infection promotes the expression of duck PIAS2 in duck cells [26]. Therefore, the expression of some PIASs in human, chicken, and duck is upregulated during influenza A virus infection. However, we also found that the expression levels of chPIAS2 induced by the same dose of DK65 were different at different time points. The possible reason is that various host proteins are involved in the regulation of chPIAS2 expression, and the underlying mechanism remains to be further investigated.

PIAS proteins interact with viral proteins and regulate their SUMOylation, thereby either inhibiting viral replication or enhancing viral infection [35]. Human PIAS1 interacts with the PB2 protein of the H1N1 influenza A virus to mediate its SUMOylation [25]. SUMOylation of PB2 reduces its stability through ubiquitination-dependent proteasomal degradation, thereby inhibiting the activity of the viral RNP complex, viral replication, and virulence in mice [25]. However, human PIAS1 also interacts with PB1 and NP of the H1N1 influenza A virus [25]. Human PIASxα interacts with the H1N1 influenza A virus NP to facilitate its SUMOylation [16]. SUMOylation of NP does not influence NP stability or viral polymerase activity but instead regulates the intracellular trafficking of NP, thereby enhancing viral production [16]. In contrast, human PIASxβ does not bind H1N1 influenza A virus NP [16]. Therefore, while human PIAS1 targets and SUMOylates the PB2 protein of the viral RNP complex to reduce PB2 stability and inhibit viral replication, human PIASxα targets and SUMOylates NP in the same complex to promote viral replication, highlighting that different PIAS proteins modulate viral production by targeting distinct subunits of the viral RNP complex. Additionally, human PIASxβ regulates SUMOylation of the hepatitis C virus core protein by interacting with it, thereby restricting viral replication [19]. Human PIASxα and PIASxβ both bind EBV Rta protein in the nucleus, but PIASxβ preferentially catalyzes its SUMOylation, activating the viral lytic cycle [18]. Duck PIAS2 interacts with H5N1 AIV NP to promote its SUMOylation and enhance viral replication [26]. Mouse PIAS4 binds the capsid protein of Moloney murine leukemia virus and mediates its SUMOylation to promote the formation of circular viral DNAs or integrated provirus during early stages of infection [36].

In this study, we found that chPIAS2 interacted with DK65-NP in the nucleus and enhanced its SUMOylation. Moreover, the SUMO E3 ligase active site of human PIAS2 (PIASxα/PIASxβ) is W383, while that of duck PIAS2 is W374 [30, 34]. In our study, we identified W383 as the SUMO E3 ligase active site of chPIAS2, which closely resembles that of human PIAS2. We further confirmed that the SUMOylation of DK65-NP transfected with the chPIAS2mut (W383A) was significantly weaker than with chPIAS2. These results suggest that chPIAS2 possesses SUMO E3 ligase activity that enhances SUMOylation of DK65-NP. Furthermore, SUMOylation is a reversible process regulated by SENPs, which play dual roles by facilitating SUMO maturation as well as deconjugating SUMO from target proteins [37, 38]. We found that SUMOylation of DK65-NP was reversible by chSENP1. Notably, in infection experiments, we also observed that overexpression of chPIAS2 promoted viral replication in DF-1 cells. Therefore, our findings indicate that chPIAS2 acts as a SUMO E3 ligase that increases SUMOylation of DK65-NP, thereby enhancing viral replication. These results also demonstrate that PIAS proteins from different species regulate viral replication and production through diverse mechanisms.

SUMOylation is a ubiquitination-like process in which SUMO covalently attaches to lysine (K) residues on target proteins or binds noncovalently to SIMs of target proteins [1]. The K4 and K7 residues in human H1N1 influenza A virus NP are SUMOylated by human PIASxα, promoting the intracellular trafficking of NP and virus growth in A549 cells [16]. In H5N1 AIV, K7, K48, and K87 residues in NP are SUMOylated by duck PIAS2, increasing viral replication in duck embryo fibroblast cells [26]. The A215T mutation abolishes the SUMOylation of H5N1 influenza A virus M1 protein, reducing viral replication in Madin–Darby canine kidney (MDCK) cells [39]. PIAS1-mediated SUMOylation of PB2 inhibits the replication and pathogenesis of influenza A virus at early time points of infection in cells and mice [25]. The K612R mutation abolishes the SUMOylation of H1N1 influenza A virus PB1 protein, significantly attenuating the pathogenicity in mice and airborne transmission among ferrets [40]. In this study, we identified K7, K48, K77, and K113 in H6N2 DK65-NP as the sites at which chPIAS2 mediated SUMOylation. Meanwhile, we found that overexpression of chPIAS2 promotes DK65 replication at 12 h in DF-1 cells. The above results indicated that these variations in SUMOylation sites affect viral replication or pathogenicity. Additionally, the interaction between flexible loop 1 (residues 73–90) of NP of influenza A virus and K113 on the adjacent β-sheet 1 (residues 91–112) is required for RNA binding, which likely involves a conformational change of NP [41]. The K7, K48, K77, and K113 sites belong to the viral polymerase-binding domain of NP of influenza A virus, and this domain interacts with both PB1 and PB2 [42]. SUMOylation of influenza A virus NP at K4 and K7 does not affect its ability and viral RNA polymerase activity, but SUMOylation directly affects the nuclear import of NP to promote virus growth in A549 cells [16]. Therefore, some SUMOylation sites of influenza A virus NP may modulate viral RNA binding of NP, viral RNA polymerase activity, and viral replication.

In conclusion, our study demonstrates that chPIAS2 interacts with the viral NP of the H6N2 AIV stain DK65 in the nucleus and promotes SUMOylation of DK65-NP at lysine residues K7, K48, K77, and K113 via its SUMO E3 ligase activity, thereby enhancing viral replication. These findings highlight the role of chPIAS2 in AIV replication and provide new insights into the molecular mechanisms underlying AIV pathogenicity in poultry.

## Data Availability

All data generated during this study are included in this published article. The raw data generated during the current study are available from the corresponding author upon reasonable request.
